# Admission Systolic Blood Pressure and Outcomes After Endovascular Thrombectomy: An International EVA-TRISP Cohort Study

**DOI:** 10.1212/WNL.0000000000214530

**Published:** 2026-01-14

**Authors:** Nabila Wali, Abris D. Mumcuoglu, Sophie A. Van Den Berg, Anne Van Der Meij, Ayesha Sajjad, Mirjam Rachel Heldner, Christina C. Marti, Petra Cimflova, Sara Magdalena Pilgram-Pastor, Marcel Arnold, Susanne Wegener, Hakim Baazaoui, Corinne Inauen, Christoph Globas, Annika Nordanstig, Patrik Michel, Georg Kägi, Christoph Riegler, Christian H. Nolte, Regina Von Rennenberg, Sami Curtze, Miranda Nybondas, Nicolas Martinez-Majander, Andrea Zini, Stefano Forlivesi, Matteo Paolucci, João Pedro Marto, Miguel Serôdio, Inês Carmo e Pinto, Alessandro Pezzini, Carlo W. Cereda, Guido Bigliardi, Gabriele Vandelli, Visnja Padjen, Tamara Svabic-Medjedovic, Issa Metanis, Ronen R. Leker, R.M. Van den Berg-Vos, Stefan T. Engelter, Henrik Gensicke, Paul J. Nederkoorn, for the EndoVAscular treatment and ThRombolysis for Ischemic Stroke Patients (EVA-TRISP) investigators

**Affiliations:** 1 Department of Neurology, Amsterdam UMC, University of Amsterdam, the Netherlands;; 2 Utch Institute for Clinical Auditing, Leiden, the Netherlands;; 3 Department of Neurology, Inselspital, Bern University Hospital and University of Bern, Switzerland;; 4 Institute of Diagnostic and Interventional Neuroradiology, Inselspital, University Hospital and University of Bern, Switzerland;; 5 Department of Neurology, University Hospital Zurich and University of Zurich, Switzerland;; 6 Department of Clinical Neuroscience, Institute of Neuroscience and Physiology, Department of Neurology, Sahlgrenska University Hospital, Sahlgrenska Academy at University of Gothenburg, Sweden;; 7 Stroke Center, Neurology Service, Lausanne University Hospital and University of Lausanne, Switzerland;; 8 Department of Neurology with Experimental Neurology, Charité–Universitätsmedizin Berlin, Corporate member of Freie Universität Berlin and Humboldt Universität zu Berlin, and Berlin Institute of Health (BIH), Germany;; 9 Neurology, University of Helsinki and Helsinki University Hospital, Finland;; 10 IRCCS Istituto delle Scienze Neurologiche di Bologna, Department of Neurology and Stroke Center, Maggiore Hospital, Italy;; 11 Department of Neurology, Hospital de Egas Moniz, Centro Hospitalar Lisboa Ocidental, Portugal;; 12 Department of Medicine and Surgery, University of Parma, Italy;; 13 Stroke Care Program, Department of Emergency, Parma University Hospital, Italy;; 14 Stroke Center and Department of Neurology, Neurocenter of Southern Switzerland, EOC, Lugano;; 15 Stroke Unit, Department of Neuroscience, Ospedale Civile di Baggiovara, Modena University Hospital, Modena, Italy;; 16 Neurology Clinic, Faculty of Medicine, University Clinical Centre of Serbia, University of Belgrade, Serbia;; 17 Department of Neurology, Hadassah-Hebrew University Medical Center, Jerusalem, Israel;; 18 Department of Neurology, OLVG, Amsterdam, the Netherlands;; 19 Neurology and Neurorehabilitation, University Department of Geriatric Medicine FELIX PLATTER, University of Basel, Switzerland; and; 20 Stroke Center and Department of Neurology, University Hospital Basel and University of Basel, Switzerland.

## Abstract

**Background and Objectives:**

Current international guidelines recommend blood pressure (BP) thresholds for patients eligible for endovascular thrombectomy (EVT). Previous studies have suggested that both low and high admission BPs are associated with poor functional outcome after EVT. However, the association between admission BP and outcomes after EVT remains poorly understood.

The aim of this study was to investigate the relationship between admission systolic BP (SBP) and outcomes in patients treated with EVT and to assess whether this association is modified by IV thrombolysis (IVT) treatment and recanalization status.

**Methods:**

In this observational, international, multicenter cohort study, we used data from the EVA-TRISP registry. Consecutive patients treated with EVT with available admission SBP were included. The primary outcome was 90-day functional outcome. Secondary outcomes included 90-day mortality, 24-hour NIH Stroke Scale (NIHSS), successful recanalization, and symptomatic intracranial hemorrhage (sICH). We used multivariable regression to study the relation between admission SBP and outcomes and to assess effect modification by IVT treatment and recanalization status.

**Results:**

We included 10.963 EVT patients. At baseline, the mean age was 72.8 years (SD 13.5), 50.2% were female and the median NIHSS at presentation was 15 (interquartile range 9–19). The association between admission SBP and functional outcome, mortality, and 24-hour NIHSS score was U-shaped, and the nadir was around 150 mm Hg. Below 150 mm Hg, every 10 mm Hg decrease in SBP was associated with higher odds of poor functional outcome (adjusted odds ratio (aOR) 1.07 [95% CI 1.02–1.11]) and mortality (aOR 1.17 [1.12–1.23]). Above 150 mm Hg, every 10 mm Hg increase in SBP was associated with higher odds of poor functional outcome (aOR 1.05 [1.01–1.08]), mortality (aOR 1.04 [1.01–1.09]), and higher 24-hour NIHSS score (β-coefficient 0.28 [0.17–0.40]). We found a positive linear relationship between admission SBP and sICH (1.04 [1.01–1.08]). IVT treatment modified the association between admission SBP and outcomes after EVT. In 5544 EVT-only treated patients, there was no longer a clear association between higher admission SBP values and worse outcome.

**Discission:**

Lower and higher admission SBP was associated with worse outcomes in the complete cohort. In EVT-only patients, this association was less evident, suggesting that high admission BP alone should not always delay or preclude treatment with EVT in otherwise eligible patients.

## Introduction

High blood pressure (BP) is common in acute ischemic stroke (AIS) patients.^1^ The exact cause of elevated BP in these patients is unclear but may represent a compensatory mechanism to increase cerebral blood flow to the ischemic area.^2^ Alternatively, it may represent preexisting poorly controlled BP or be secondary to the stress experienced because of the stroke. Current guidelines recommend BP thresholds for patients eligible for endovascular thrombectomy (EVT). The European Stroke Organization recommends BP below 180/105 mm Hg during EVT,^3^ and the American Heart Association recommends maintaining BP below 185/110 mm Hg before EVT.^4^ These recommendations, however, are based on the threshold recommended for treatment with IV thrombolysis (IVT), which was established to reduce the risk of intracerebral hemorrhage associated with IVT. The BP threshold for IVT eligible patients was mainly based on a clinical trial conducted in the 1990s, which based the threshold on expert consensus and results from smaller cohort studies.^5^ Since clinical EVT trials excluded patients with a BP above 185/110 mm Hg, the BP cutoff for IVT patients was later arbitrarily adopted for EVT patients.

In previous observational studies, both high and low admission BPs have been associated with worse outcomes in AIS patients.^6-9^ A patient-level meta-analysis of 7 randomized controlled trials showed that high admission systolic BP (SBP) was associated with worse functional outcomes, but high BP did not reduce the positive effects of EVT.^10^ Although observational studies have shown a relationship between high admission BP and worse outcomes after EVT, other studies have shown that active BP lowering in AIS patients does not improve outcomes and may even lead to worse outcomes.^11-16^ As a result, there is still no consensus about whether to lower BP actively or withhold or delay EVT in patients with high BP who are otherwise eligible for EVT. These conflicting findings show the complexity of the relationship between BP and outcomes after EVT.

This study aims to further investigate the relationship between admission SBP and outcomes in patients treated with EVT in a large observational cohort and to assess whether this association is modified by treatment modality (treatment with EVT and IVT or EVT-only) and recanalization status.

## Methods

### Study Design and Patient Selection

This was a multicenter cohort study from the EndoVAscular treatment and ThRombolysis for Ischemic Stroke Patients (EVA-TRISP) collaboration, including data from 16 participating centers. The methodology of the EVA-TRISP has been described previously.^17^ To briefly summarize, the EVA-TRISP collaboration aims to investigate the safety and outcome of EVT outside the clinical trial setting by prospectively collecting predefined data on EVT patients. The collaboration includes dedicated stroke centers, mainly across Europe, with expertise in EVT.

In this study, consecutive EVT patients between 2013 and 2023 were included. The inclusion period varied across the different participating centers (eTable1). Patients who met the following criteria were included in the analyses: large vessel occlusion in the proximal anterior circulation (i.e., the intracranial internal carotid artery, the middle cerebral artery [M1/M2], or the anterior cerebral artery [A1/A2]), as confirmed by CT angiography, magnetic resonance angiography, or digital subtraction angiography. Patients with missing admission SBP values, as well as patients younger than 18 years, were excluded from analyses. Admission BP was defined as the first measured SBP recorded on the patient's arrival at the emergency department, typically measured noninvasively with an automated sphygmomanometer.

### Outcomes

The primary outcome measure was the dichotomized modified Rankin Scale (mRS) score at 90 days. A good functional outcome was defined as a mRS score between 0 and 2, while a poor functional outcome was defined as a mRS score between 3 and 6. The mRS score at 90 days was assessed by telephone interviews, postal or electronic questionnaires, or outpatient visits. Relatives or health personnel close to the patient were asked when it was not possible to obtain the mRS score from the patient. Secondary outcome measures included 90-day mortality, 24-hour NIH Stroke Scale (NIHSS) score, symptomatic intracranial hemorrhage (sICH), and rates of successful recanalization. Recanalization status was assessed during EVT on the final angiogram. Successful recanalization was defined as a (modified) treatment in cerebral infarction ([m] TICI) score of 2b or higher. sICH was defined according to the European Cooperative Acute Stroke Study II criteria^18^: any intracranial hemorrhage detected on follow-up imaging after treatment, accompanied by clinical deterioration resulting in a ≥4 point increase in the NIHSS score within 7 days after stroke onset.

### Statistical Analysis

To evaluate potential nonlinear relationships between admission SBP and outcome variables, we compared an unadjusted linear model for admission SBP with a quadratic and a restricted cubic spline model. This comparison was assessed using the likelihood ratio test. Missing covariate data were addressed using multiple imputations by chained equations (n = 10) to estimate missing values.

We used mixed linear and logistic regression incorporating stroke center as a random effect. Potential confounders were identified with the use of a directed acyclic graph (eFigure 1). We adjusted for age, history of hypertension, time from last seen well or onset to groin puncture, and NIHSS score on admission as fixed effects in the regression models. Unadjusted and adjusted predicted probabilities along with their corresponding CIs were calculated and plotted against admission SBP.

For outcomes with a nonlinear association with SBP, the SBP value at the inflection point of the outcome variable was selected as a reference point to divide the cohort, to subsequently investigate outcomes separately for the lower and higher SBP groups. Outcomes with a linear relation to admission SBP were analyzed across the complete cohort. Unadjusted and adjusted odds ratios (ORs) or beta-coefficients with their corresponding 95% CIs were calculated per 10 mm Hg decrease below the inflection point for the lower admission SBP (below 150 mm Hg) group and per 10 mm Hg increase above the inflection point for the higher admission SBP (above 150 mm Hg) group.

Baseline demographics, clinical characteristics, and outcomes were compared using the unpaired *t* test or the Mann-Whitney *U* test for continuous variables and X2 test for categorical variables. A *p* value ≤0.05 was considered statistically significant.

To investigate whether treatment modality and recanalization status affected the association between admission SBP and the outcomes, we constructed interaction terms with treatment modality and recanalization status and performed stratified analyses. We categorized treatment modality as treatment with EVT and IVT and treatment with EVT-only. We categorized recanalization status as successful recanalization (mTICI 2b-3) and unsuccessful recanalization (mTICI 0-2a).

All statistical analyses were performed in IBM SPSS statistics (version 28) and R software (version 4.4.0).

### Standard Protocol Approvals, Registrations, and Patient Consents

Approval from local authorities and/or ethical committees was obtained by each treatment center in compliance with national and local regulations and requirements. Owing to the observational nature of the study, the requirement for informed consent was waived or an opt-out procedure was available at the participating centers.

### Data Availability

The individual deidentified data from the EVA-TRISP cohort supporting the findings of this study are not publicly available because of ethical regulations and agreements within the EVA-TRISP collaboration. Data may be made available on reasonable request to the EVA-TRISP collaboration. Requests are subject to approval by the contributing centers.

## Results

### Baseline Characteristics and Outcomes of the Complete Cohort

Of the 14,618 consecutive patients in the EVA-TRISP cohort, 10,963 patients from 16 EVT centers were included in the current analyses (eFigure 2). In eTable 1, the number of patients per center is depicted.

Mean admission SBP was 152 mm Hg (SD 26.9), and mean admission diastolic BP was 83 mm Hg (17.5). Mean age was 72.8 years (SD 13.5), and 5,456 (49.8%) patients were male. In total, 7,423 (67.8%) patients had a medical history of hypertension, 9,336 (89.1%) patients had a prestroke mRS score between 0 and 2, and median NIHSS at admission was 15 (interquartile range [IQR] 9–19). A total of 5,417 (49.4%) patients received treatment with IVT in addition to EVT (Table 1).

**Table 1:** Baseline Characteristics Complete Cohort and Lower and Higher SBP Groups

	All patients n = 10,963	Admission SBP ≤150 mm Hg n = 5,672	Admission SBP >150 mm Hg n = 5,291	*p* Value
Age in y, mean (SD)	72.8 (13.5)	70.4 (14.4)	75.3 (11.8)	<0.001
Sex male, n (%)	5,456 (49.8)	2,879 (50.8)	2,577 (48.8)	0.033
Missing, n (%)	10 (0.1)	5 (0.1)	5 (0.1)	
Admission SBP in mm Hg, mean (SD)	151.9 (26.9)	131.3 (14.8)	173.9 (18.2)	<0.001
Admission DBP in mm Hg, mean (SD)	83.0 (17.5)	76.4 (14.3)	90.1 (18.0)	<0.001
Missing, n (%)	10 (0.1)	4 (0.1)	6 (0.1)	
Medical history, n (%)				
Atrial fibrillation	4,109 (37.5)	2025 (35.7)	2084 (39.4)	<0.001
Missing, n (%)	14 (0.1)	6 (0.1)	8 (0.2)	
Diabetes mellitus	2032 (18.6)	973 (17.2)	1,059 (20.0)	<0.001
Missing, n (%)	9 (0.1)	6 (0.1)	3 (0.1)	
Hypertension	7,423 (67.8)	3,474 (61.3)	3,949 (74.7)	<0.001
Missing, n (%)	14 (0.1)	7 (0.1)	7 (0.1)	
Hypercholesterolemia	5,271 (48.1)	2,631 (46.4)	2,640 (50.0)	<0.001
Missing, n (%)	13 (0.1)	6 (0.1)	7 (0.1)	
Coronary heart disease	1820 (18.0)	940 (18.1)	880 (17.8)	0.743
Missing, n (%)	833 (7.6)	478 (8.4)	355 (6.7)	
Prior ischemic stroke	1,455 (14.0)	754 (14.1)	701 (13.9)	0.821
Missing, n (%)	583 (5.3)	325 (5.7)	258 (4.9)	
Current smoking (or stopped <2 y ago)	1993 (19.2)	1,164 (21.6)	829 (16.7)	<0.001
Missing, n (%)	603 (5.5)	283 (5.0)	320 (6.0)	
Independent prestroke^a^, n (%)	9,336 (89.1)	4,821 (88.9)	4,515 (89.3)	0.605
Missing, n (%)	486 (4.4)	252 (4.4)	234 (4.4)	
NIHSS at presentation, median (IQR)	15 (9–19)	15 (9–19)	14 (9–19)	0.204
Missing, n (%)	146 (1.3)	85 (1.5)	61 (1.2)	
Transferred from another hospital, n (%)	4,321 (40.0)	2,323 (41.7)	1998 (38.2)	<0.001
Missing, n (%)	164 (1.5)	100 (1.8)	64 (1.2)	
Treatment with IVT, n (%)	5,417 (49.4)	2,807 (49.5)	2,610 (49.3)	0.619
Missing, n (%)	1 (0.0)	0 (0.0)	1 (0.0)	
Onset of symptoms to groin puncture time in minutes, median (IQR)	198 (151–280)	195 (150–275)	200 (155–280)	0.012
LSW to groin puncture time in minutes, median (IQR)	255 (165–495)	252 (162–490)	257 (170–503)	0.035
Onset/LSW to groin puncture time, median (IQR)	228 (160–390)	222 (155–379)	235 (163–405)	<0.001
Missing, n (%)	767 (7.0)	446 (7.9)	321 (6.1)	
Location of occlusion, n (%)				
ICA	2,342 (21.4)	1,146 (20.2)	1,196 (22.6)	
M1	5,466 (49.9)	2,960 (52.2)	2,506 (47.4)	
M2	3,025 (27.6)	1,532 (27.0)	1,493 (28.2)	
ACA	197 (1.8)	100 (1.8)	97 (1.8)	

Abbreviations: ACA = anterior cerebral artery; DBP = diastolic blood pressure; ICA = internal carotid artery; IQR = interquartile range; IVT = IV thrombolysis; LSW = last seen well; M1= M1 segment of the middle cerebral artery; M2 = M2 segment of the middle cerebral artery; mRS = modified Rankin Scale; NIHSS = NIH Stroke Scale; SBP = systolic blood pressure.

Depicted *p* values are for the difference between the below 150 mm Hg and the above 150 mm Hg group.

*p* values below 0.05 were considered statistically significant.

a Defined as a modified Rankin scale score = 0–2.

In the complete cohort, 5,622 (55.9%) patients had a poor functional outcome 3 months after the ischemic stroke. Successful recanalization was achieved in 6,363 (73.1%) patients, sICH occurred in 475 (5.2%) patients, and median 24-hour NIHSS score after EVT was 8 (IQR 3–16) (Table 2).

**Table 2:** Outcomes Complete Cohort and Lower and Higher SBP Groups

	All patients n = 10.963	Admission SBP ≤150 mm Hg n = 5,672	Admission SBP >150 mm Hg n = 5,291	*p* Value
mRS at 90 d, %				<0.001
0	1,174 (11.7)	647 (12.5)	527 (10.8)	
1	1,670 (16.6)	875 (16.9)	795 (16.3)	
2	1,593 (15.8)	877 (16.9)	716 (14.7)	
3	1,500 (14.9)	761 (14.7)	739 (15.1)	
4	1,305 (13.0)	656 (12.7)	649 (13.3)	
5	487 (4.8)	227 (4.4)	260 (5.3)	
6	2,330 (23.2)	1,137 (21.9)	1,193 (24.5)	
Missing, n (%)	904 (8.2)	492 (8.7)	412 (7.8)	
Poor functional outcome at 90 d^a^, n (%)	5,622 (55.9)	2,781 (53.7)	2,841 (58.2)	<0.001
Mortality at 90 d^b^, n (%)	2,330 (23.2)	1,137 (21.9)	1,193 (24.5)	0.003
sICH^c^, n (%)	475 (5.2)	223 (4.7)	252 (5.6)	0.054
Missing, n (%)	1762 (16.1)	946 (16.7)	816 (15.4)	
Successful reperfusion on DSA^d^, n (%)	6,363 (73.1)	3,325 (73.6)	3,038 (72.6)	0.311
Missing, n (%)	2,257 (20.6)	1,152 (20.3)	1,105 (20.9)	
24-h NIHSS, median (IQR)	8 (3–16)	8 (3–15)	9 (3–17)	<0.001
Missing, n (%)	1,312 (12.0)	721 (12.7)	591 (11.2)	

Abbreviations: DSA = digital subtraction angiography; mRS = modified Rankin Scale; NIHSS = NIH Stroke Scale; SBP = systolic blood pressure; sICH = symptomatic intracranial hemorrhage.

Depicted *p* values are for the difference between the below 150 mm Hg and the above 150 mm Hg group.

*p* values below 0.05 were considered statistically significant.

a Defined as a modified Rankin scale score = 0–2, 3 months after stroke onset.

b Defined as a mRS score = 6, 3 months after stroke onset.

c According to ECASS II criteria.

d Defined as a modified Thrombolysis in Cerebral Infarction Score = 2b-3.

The use of restricted cubic splines with 3 knots provided a better fit for the data in logistic regression analysis compared with a model with a linear term or a quadratic term (*p* < 0.001), indicating a nonlinear relationship between admission SBP and functional outcome, mortality, and 24-hour NIHSS score. The number of knots was determined by the Akaike Information Criterion ^19^(AIC). Knots were placed at the 10th, 50th, and 90th percentile, as determined automatically by the statistical software.

**Figure 1:**
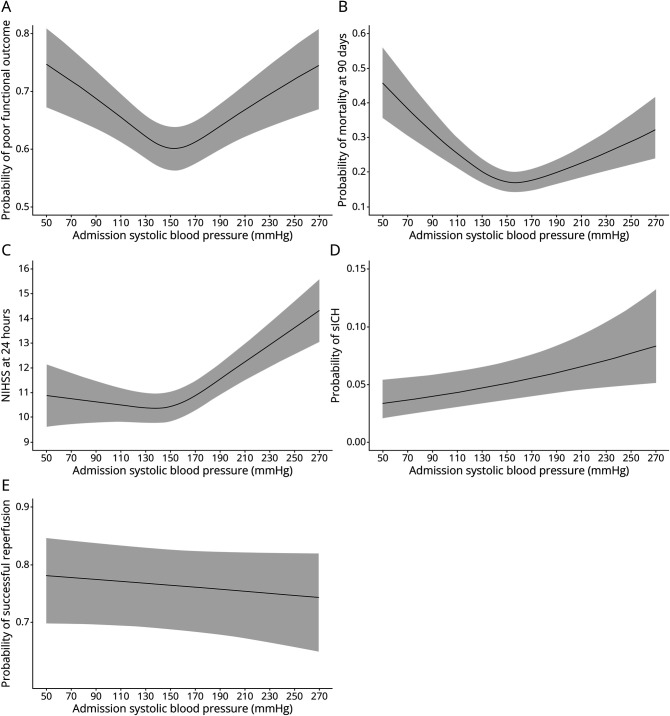
Relationship Between Admission SBP and the Outcome Variables (A) Predicted probability of poor functional outcome (mRS of 3-6 at 3 months). (B) Predicted probability of mortality at 3 months. (C) Predicted NIHSS at 24 hours. (D) Predicted probability of sICH E: Predicted probability of succesful reperfusion (mTICI 2b or higher). Adjusted for stroke center as a random effect and adjusted for age, history of hypertension, time from last seen well or onset to groin puncture, and NIHSS score on admission as fixed effects. mRS = modified rankin scale; mTICI = (modified) treatment in cerebral infarction; NIHSS = NIH Stroke Scale; SBP = systolic blood pressure; sICH = symptomatic intracranial hemorrhage.

The relationships between admission SBP and functional outcome and mortality were U-shaped, and both lower and higher admission SBPs were associated with a higher probability of worse outcomes (Figure 1, A and B). For admission SBP, the lowest predicted probability (i.e., specific inflection point) of poor functional outcome was at 153 mm Hg and for mortality at 157 mm Hg. The relationship between admission SBP and 24-hour NIHSS score followed a J-shape, and mainly higher BPs were associated with an increased probability of higher 24-hour NIHSS scores. The inflection point was at 137 mm Hg. We observed a positive linear relationship between admission SBP and sICH and a negative linear relationship for successful recanalization (with broad CIs) (Figure 1, D and E).

### Baseline Characteristics and Outcomes for Lower and Higher Admission BP Groups

**Figure 2:**
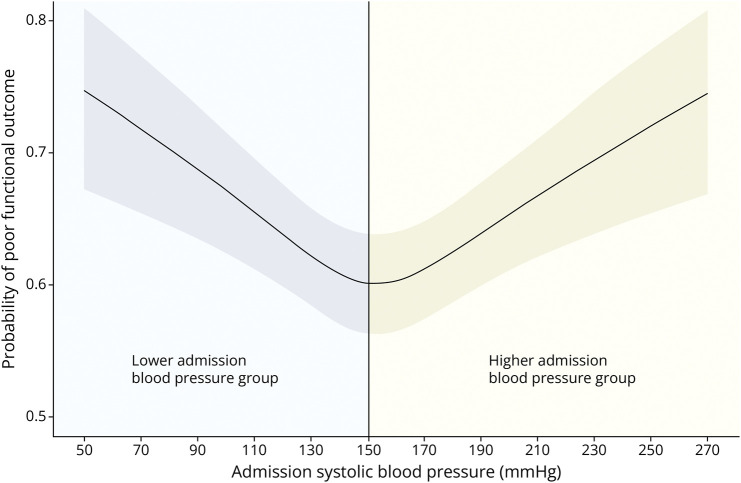
Division of the Cohort for Analysis of Lower and Higher Blood Pressure Groups Separately The same figure as Figure 1A is depicted here, this figure depicts how the cohort was divided into 2 groups for separate analysis.

The inflection points of the relationship between BP and poor functional outcome, mortality, and 24-hour NIHSS were approximately 150 mm Hg (the median SBP value); this cutoff was chosen to divide the cohort into a lower admission SBP (≤150 mm Hg) group and a higher admission SBP (>150 mm Hg) group (Figure 2). Regression analysis was then performed separately for these groups.

Compared with the 5,672 patients in the lower admission SBP group, the 5,291 patients in the higher admission SBP group were older (75.3 [SD 11.8] vs 70.4 years [SD 14.4]) and more often had a medical history of hypertension, diabetes, and hypercholesterolemia. Patients in the higher admission SBP group also had a longer onset or last seen well to groin time (median 235 [IQR 163–405] vs 222 minutes [IQR 155–379]) (Table 1).

Patients in the higher admission SBP group had higher rates of poor functional outcome (2,841 [58.2%] vs 2,781 [53.7%]), higher mortality rates (1,193 [24.5%] vs 1,137 [21.9%]), and higher median 24-hour NIHSS scores after EVT (median 9 [IQR 3–17] vs 8 [IQR 3–15]) compared with the lower SBP group (Table 2).

In the multivariable mixed regression analysis, patients in the lower admission SBP group had significantly higher odds of poor functional outcome (adjusted OR 1.07 [95% CI 1.03–1.09], per 10 mm Hg decrease, *p* value 0.002) and mortality (adjusted OR 1.17 [95% CI 1.12–1.13], per 10 mm Hg decrease, *p* value <0.001). In the higher admission SBP group, every 10 mm Hg increase in BP was associated with significantly higher odds of poor functional outcome (adjusted OR 1.05 [95% CI 1.01–1.08], *p* value 0.0129), mortality (adjusted OR 1.04 [95% CI 1.01–1.09], *p* value 0.026), and 24-hour NIHSS score (adjusted β-coefficient 0.28 [95% CI 0.17–0.40], *p* value <0.001). The association between admission SBP and sICH was linear, and every 10 mm Hg increase in admission SBP was associated with higher odds of sICH (adjusted OR 1.04 [95% CI 1.01–1.08], *p* value 0.015) (Table 3).

**Table 3:** Regression Analyses Complete Cohort

	SBP ≤150 mm Hg				SBP >150 mm Hg			
	Unadjusted OR	*p* Value	Adjusted OR	*P* Value	Unadjusted OR	*p* Value	Adjusted OR	*P* Value
Poor functional outcome at 90 d^a^	1.03 (1.00–1.07)	0.082	1.07 (1.02–1.11)	0.002	1.06 (1.03–1.09)	<0.001	1.05 (1.01–1.08)	0.013
Mortality at 90 d^b^	1.14 (1.10–1.19)	<0.001	1.17 (1.12–1.23)	<0.001	1.06 (1.03–1.10)	<0.001	1.04 (1.01–1.09)	0.026
24-h NIHSS	0.18 (0.03–0.35)	0.022	0.08 (−0.06 to 0.22)	0.251	0.27 (0.13–0.41)	<0.001	0.28 (0.17–0.40)	<0.001
	SBP continuous							
sICH^c^	1.04 (1.01–1.08)	0.016	1.04 (1.01–1.08)	0.015				
Successful reperfusion on DSA^d^	0.99 (0.97–1.01)	0.309	0.99 (0.97–1.01)	0.206				

Abbreviations: DSA = digital subtraction angiography; NIHSS = NIH Stroke Scale; OR = odds ratio; SBP = systolic blood pressure; sICH = symptomatic intracranial hemorrhage.

Adjusted for stroke center as a random effect and adjusted for age, history of hypertension, time from last seen well or onset to groin puncture, and NIHSS score on admission as fixed effects.

All odds ratios are depicted with their 95% CIs between brackets.

*p* values below 0.05 were considered statistically significant.

a Defined as a modified Rankin scale score = 0–2, 3 months after stroke onset.

b Defined as a mRS score = 6, 3 months after stroke onset.

c According to ECASS II criteria.

d Defined as a modified Thrombolysis in Cerebral Infarction Score = 2b-3.

### EVT-Only Subgroup

We found statistically significant interactions between admission SBP and treatment modality in the association between poor functional outcome, mortality, and 24-hour NIHSS score (*p* values <0.001). We found statistically significant interactions between admission SBP and recanalization status for all outcome variables (*p* values <0.001). Since we observed substantial interactions, we stratified the cohort by treatment modality (EVT treated patients only, no IVT) and by recanalization status (successfully recanalized patients only). We then performed subgroup analyses to further explore the association between admission SBP and outcomes in these groups.

In a subgroup analysis of 5,544 patients treated with EVT-only (without prior IVT), patients more often had atrial fibrillation (2,409 (43.5%) vs 4,109 (37.5%), a lower median NIHSS score at admission (median 14 (IQR 9–19) vs 15 (IQR 9–19) and longer onset or last seen well to groin times (median 315 minutes [IQR 184–641] vs median 228 minutes [IQR 160–390]) at baseline in comparison with the complete cohort. No other major differences at baseline were observed between the EVT-only group and the complete cohort (Table 1 and eTable 2).

In the EVT-only group, 3,181 (62.5%) patients had poor functional outcome 3 months after the ischemic stroke, successful recanalization was achieved in 3,195 (72.7%) patients, sICH occurred in 240 (5.0%) patients, and median 24-hour NIHSS was 9 (IQR 4–17) (eTable 2).

**Figure 3:**
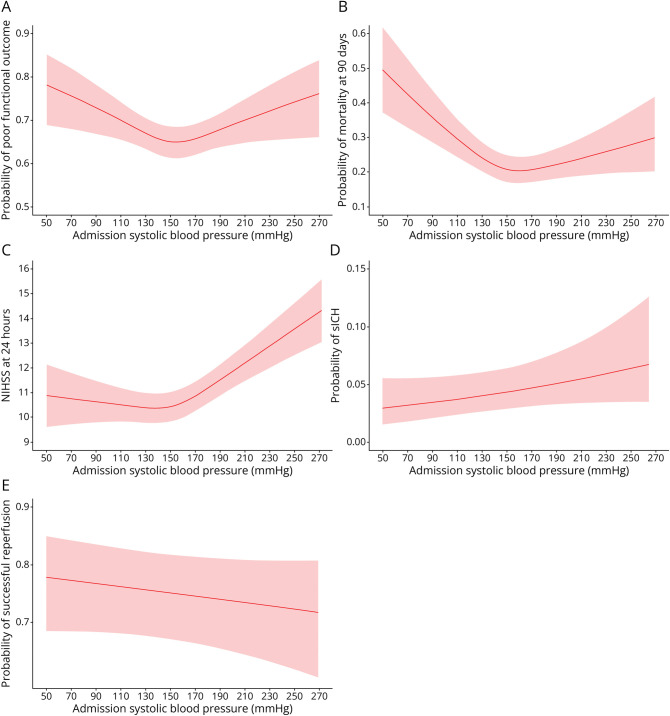
Relationship Between Admission SBP and the Outcome Variables in the EVT-Only Subgroup (A) Predicted probability of poor functional outcome (mRS of 3-6 at 3 months). (B) Predicted probability of mortality at 3 months. (C) Predicted NIHSS at 24 hours. (D) Predicted probability of sICH. (E) Predicted probability of succesful reperfusion (mTICI 2b or higher). Adjusted for stroke center as a random effect and adjusted for age, history of hypertension, time from last seen well or onset to groin puncture, and NIHSS score on admission as fixed effects. EVT = endovascular thrombectomy; mRS = modified rankin scale; NIHSS = NIH Stroke Scale; SBP = systolic blood pressure; sICH = symptomatic intracranial hemorrhage.

In this subgroup, the relationship between admission SBP and our outcome variables showed similar patterns in the (cubic spline) curves as observed in the whole cohort (Figure 3). However, although SBPs lower than 150 mm Hg were associated with increased odds of poor functional outcome (adjusted OR 1.07 [95% CI 1.01–1.13], *p* value 0.022) and mortality (adjusted OR 1.16 [95% CI 1.10–1.23], *p* value <0.001). By contrast, for SBP values above 150 mm Hg, the associations with all of the outcome variables were no longer statistically significant (Table 4).

**Table 4:** Regression Analyses EVT-Only Subgroup

	SBP ≤150 mm Hg				SBP >150 mm Hg			
	Unadjusted OR	*p* Value	Adjusted OR	*P* Value	Unadjusted OR	*p* Value	Adjusted OR	*p* Value
Poor functional outcome at 90 d^a^	1.04 (0.99–1.10)	0.110	1.07 (1.01–1.13)	0.022	1.02 (0.98–1.07)	0.275	1.02 (0.97–1.08)	0.356
Mortality at 90 d^b^	1.15 (1.09–1.21)	<0.001	1.16 (1.10–1.23)	<0.001	1.02 (0.97–1.07)	0.379	1.01 (0.96–1.06)	0.637
24-h NIHSS	0.24 (0.02–0.46)	0.036	0.12 (−0.07 to 0.31)	0.216	0.08 (−0.11 to 0.27)	0.399	0.16 (−0.01 to 0.32)	0.063
	SBP continuous							
sICH^c^	1.02 (0.97–1.07)	0.454	1.04 (0.99–1.09)	0.140				
Successful reperfusion on DSA^d^	0.99 (0.96–1.01)	0.214	0.98 (0.95–1.01)	0.115				

Abbreviations: DSA = digital subtraction angiography; NIHSS = NIH Stroke Scale; OR = odds ratio; SBP = systolic blood pressure; sICH = symptomatic intracranial hemorrhage.

Adjusted for stroke center as a random effect and adjusted for age, history of hypertension, time from last seen well or onset to groin puncture, and NIHSS score on admission as fixed effects.

All odds ratios are depicted with their 95% CIs between brackets.

*p* values below 0.05 were considered statistically significant.

a Defined as a modified Rankin scale score = 0–2, 3 months after stroke onset.

b Defined as a mRS score = 6, 3 months after stroke onset.

c According to ECASS II criteria.

d Defined as a modified Thrombolysis in Cerebral Infarction Score = 2b-3.

### Successful Recanalization Subgroup

In the successful recanalization subgroup, no clear differences at baseline were observed in comparison with the complete cohort (Table 1 and eTable 3). In this subgroup, the cubic spline and linear curves also showed similar patterns for all outcome variables, as observed in the complete cohort. However, the curves shifted toward better outcomes, indicating an overall higher likelihood of better outcomes in the successful reperfusion subgroup (eFigure 3).

In the lower admission SBP (below 150 mm Hg) group of the successful recanalization subgroup, every 10 mm Hg decrease in SBP was associated with higher odds of mortality (adjusted OR 1.19 [95% CI 1.12–1.27], *p* value <0.001). In the higher admission SBP group (above 150 mm Hg), every 10 mm Hg increase in SBP was associated with higher odds of 90-day poor functional outcome (adjusted OR 1.07 [1.02–1.12], *p* value 0.004) and higher 24-hour NIHSS (adjusted β-coefficient 0.31 [95% CI 0.16–0.46], *p* value <0.001) (eTable 4).

## Discussion

In this large, prospective, multicenter cohort of consecutive patients treated with EVT, both lower and higher admission SBPs were associated with higher rates of poor functional outcome and mortality when analyzing the complete cohort. In the complete cohort, higher admission SBP was associated with higher rates of sICH and higher 24-hour NIHSS scores. Treatment type (EVT and IVT or EVT-only) and recanalization status modified the association between admission SBP and outcomes. Of interest, when we stratified the cohort based on treatment type, the association between higher admission SBP and worse outcomes was not evident in the subgroup of patients treated with EVT-only.

Several studies have reported U-shaped or J-shaped relationships between admission BP and outcomes in general populations of ischemic stroke or those treated with IVT or EVT.^6-9^ Although these studies show that both lower and higher SBPs are associated with poorer outcomes, the location of the inflection point (the “best” BP value) differed. In addition, in some studies, SBP was associated with an increased risk of sICH in a linear fashion,^7,8^ and we found similar results in our analyses. Not all studies investigating these associations have found statistically significant results, which could be due to a lack of power resulting from small study groups or a low occurrence of sICH in the data set. In addition, some studies report that high SBP is associated with lower odds of successful reperfusion.^20,21^ It is hypothesized that high BP could create hydraulic forces that make mechanic cloth removal more difficult.^20^ However, unlike the findings in these studies, we found no association between SBP and successful recanalization in our cohort.

The influence of admission SBP and the effect of EVT was investigated in a pooled individual patient-level meta-analysis.^10^ In this study, 1753 patients were included, 867 EVT treated patients and 886 controls. In line with our findings, a nonlinear relationship between admission SBP and functional outcome was found with a slightly lower inflection point at 140 mm Hg. Higher admission SBP was associated with worse functional outcome, higher rates of 90-day mortality, higher 24-hour NIHSS, and larger follow-up infarct volume. However, no statistically significant interaction between SBP and the effect of EVT was found, suggesting that admission SBP did not negate the effects of EVT.

Many factors influence the association between BP and outcomes in EVT, making this association complex and still not well understood. In the EVA-TRISP cohort, we found that both lower and higher admission SBPs were associated with worse outcomes in the complete cohort. Since it is hypothesized that the increase in BP in AIS patients is a compensatory mechanism to induce cerebral blood flow, low BP in these AIS patients might be due to other underlying causes. A previous study found that low SBP in AIS patients was associated with heart failure, gastrointestinal bleeding, and sepsis.^22^ It is possible that the association between low admission SBP and poor functional outcome and mortality in the EVA-TRISP cohort is explained by underlying causes rather than the AIS itself. Periprocedural low BP during EVT is often attributed to the use of sedation. Studies found that conscious sedation and general anesthesia were associated with lower BP during EVT and worse outcomes.^23-25^ In EVA-TRISP, the different treatment centers used different sedation approaches. However, we could not analyze this on a patient level. It is also suggested that BP variability in the first few days after AIS onset is also associated with worse outcomes in EVT patients.^26-28^ The hypothesis is that due to impaired autoregulation in AIS patients, BP fluctuations may compromise cerebral blood flow, especially in patients with poor collateral flow or a large infarct size.^29-31^

In EVA-TRISP, we found that recanalization status was an effect modifier in the association between admission SBP and outcomes after EVT. Although we found an association between low SBP and mortality and high SBP with poor functional outcome and higher 24-hour NIHSS scores in recanalized patients, when successful recanalization was achieved, the likelihood of overall better outcome was higher. It is suggested that the mechanism in which high BP is linked to worse outcomes is different between recanalized and nonrecanalized patients.^32^ In nonrecanalized patients, high BP may reflect a more severe stroke and the physiologic response to increase cerebral perfusion. While in recanalized patients, high BP may lead to worse outcomes due to hyperperfusion, cerebral edema, and hemorrhagic transformation. Several earlier studies also reported that recanalization status modulates the association between BP and outcomes after EVT.^33-35^

Although we found a clear association between high admission SBP and outcomes, this association was no longer evident in the subgroup analysis of 5544 EVT-only treated patients. Of interest, this finding could not be explained by the occurrence of more sICH in the group of patients also treated with thrombolytic therapy. In contrast to our findings, 2 other studies did not find an interaction between treatment with IVT and the BP and outcomes association.^36,37^ Both of these trials used data from randomized trials, and possibly the patient selection criteria in the trial setting differed from those used in routine clinical practice.

It seems reasonable to actively lower BP in EVT patients due to the association between higher BP and higher rates of sICH and poor functional outcome found in previous smaller EVT cohorts, an association we also found in the complete EVA-TRISP cohort. However, this association was not observed in the EVT-only group. Therefore, the optimal BP treatment strategy in the acute phase before EVT remains uncertain. Furthermore, caution is warranted with active BP-lowering strategies because there is growing evidence that active BP lowering before and after reperfusion therapy may lead to worse outcomes.^11-16^ A subgroup analysis of the INTERACT-4 trial showed that prehospital intensive BP lowering led to worse functional outcome compared with usual care.^14^ The Multicentre Randomised trial of Acute Stroke treatment in the Ambulance with a nitroglycerin Patch (MR ASAP) trial showed that despite a modest lowering of the SBP, transdermal ambulance delivered glyceryl trinitrate did not benefit AIS patients.^15^ Another observational study, the Thrombolysis and Uncontrolled Hypertension (TRUTH) study, investigated the effect of active BP lowering in IVT patients.^16^ In this study, standard BP-lowering strategies were compared; active BP lowering below 185/110 mm Hg or a strategy where IVT was only administered if BP declined spontaneously. A shift was observed toward worse functional outcome in the active BP-lowering group, although the finding was not statistically significant. Although there is a lack of randomized trials investigating the effect of active BP lowering before EVT, some trials have investigated the effect of active BP strategies after EVT. These trials, however, found nonsuperior or worse outcomes in patients with actively lowered BPs.^11-13^ The findings from all these studies suggest that active BP-lowering techniques should be considered cautiously in AIS patients. In EVA-TRISP, we lacked data on patient-level active BP-lowering strategies, which prevented us from drawing conclusions about the effect of active BP lowering on the association between BP and outcomes. Nonetheless, there were centers that did not consistently actively lower BP in patients who were eligible for EVT-only. Therefore, it is a possibility that fewer patients with a high BP in the EVT-only group received active BP-lowering therapy, which may explain the better outcome in the EVT-only subgroup. All in all, the association between BP and outcomes after EVT is still not fully understood, and there are conflicting results across earlier studies. In our study, we found that the association was modified by treatment type and recanalization status. Notably, in the EVT-only group, no clear association was observed between BP and worse outcomes. Given that the BP threshold for EVT in current guidelines is based on limited evidence, our results suggest that elevated BP alone should probably not delay or preclude EVT in otherwise eligible patients. Moreover, a more individualized approach to BP management is warranted. Although our findings are purely observational, which includes the potential risk of bias, they might be of interest for future guidelines on this topic, in particular because key observations deviate from recommendations in current guidelines.

There were several limitations to our study. One important limitation was that in EVA-TRISP, only a single BP value at admission was available. Owing to this, we were unable to assess the effect of potential BP fluctuations during and after the EVT procedure. Second, the EVT centers included in EVA-TRISP could have used different BP treatment strategies before and after EVT. The BP values we studied were the first SBP values recorded at admission and before potential BP-lowering treatments. However, it is possible that the BP was lowered before or after EVT treatment. Although we had information on the use of standard BP management strategies at the hospital level before IVT and EVT, in our cohort, we did not have patient-level data on active BP-lowering strategies. Therefore, we were unable to analyze the effect of active BP-lowering strategies. To account for the different treatment strategies in the centers, we used the EVT center as a random effect in the regression models. Another limitation of our study was that only EVT-treated patients were included in EVA-TRISP. Therefore, we had no data on the group of patients who did not undergo treatment with EVT due to a high BP. Precluding comparison analysis between EVT treated and non-EVT treated patients with a high BP. Owing to the real world setting of this study, the mRS and mTICI scores were assessed locally without central adjudication which may have introduced some degree of interobserver variability. However, the large number of centers and patients will in our opinion limit this effect. Furthermore, although we imputed our covariates, the outcome variables were not imputed, and therefore, a percentage of patients were excluded in regression analysis because of missing outcome variables.

One of the strengths of this study was its large cohort size, which allowed us to test for effect modification by IVT treatment and recanalization status and to investigate the association between admission SBP and outcomes in a substantial subgroup of EVT-only patients, a subgroup for which there is limited prior evidence. Other strengths of this study were its multicenter nature with participation from dedicated centers from different countries, overall good data completeness, and the inclusion of patients outside of a trial setting from routine clinical practice. Since the BP threshold before EVT is based on limited evidence, sometimes treatment was performed above guideline BP when the expected benefits were judged to outweigh the risks. This allowed us to explore outcomes across a wide BP spectrum.

In conclusion, in this large EVT cohort, both lower and higher admission SBP values were associated with worse functional outcome and mortality. Higher admission SBP was also associated with higher 24-hour NIHSS scores and higher odds of sICH. However, in the subgroup of patients treated solely with EVT (no IVT), higher admission BP was not associated with worse outcome. These findings suggest that increased admission BP should not always delay or preclude treatment with EVT, thereby supporting broader treatment eligibility in clinical practice.

## Glossary

(m) TICI: (modified) treatment in cerebral infarction
AIS: acute ischemic stroke
BP: blood pressure
EVT: endovascular thrombectomy
IQR: interquartile range
IVT: IV thrombolysis
mRS: modified Rankin Scale
NIHSS: NIH Stroke Scale
OR: odds ratio
SBP: systolic BP
sICH: symptomatic intracranial hemorrhage
